# From capacity to value: reframing health-system reform in Kuwait

**DOI:** 10.3389/frhs.2026.1991280

**Published:** 2026-10-29

**Authors:** Abdulrahman Alqashaan, Sultan E. Alsalahi, Abdulaziz Alhenaidi, Hisham Kelendar

**Affiliations:** Ministry of Health, Kuwait City, Kuwait

**Keywords:** Gulf Cooperation Council, health financing, health governance, health information systems, health systems, Kuwait, value-based healthcare

## Introduction

Over the past century, Kuwait has developed a health system encompassing all six components the World Health Organization (WHO) identifies as essential: service delivery, health workforce, health information systems, medical products and technologies, financing, and leadership and governance (1). National data from 2011 to 2022 show substantial expansion and resilience amid global shocks (2), and Kuwait compares favourably on key outcomes, with a life expectancy of approximately 80 years and an under-five mortality rate of 8.56 per 1,000 live births (3). The central policy question is therefore not whether Kuwait has capacity, but how that capacity is governed, measured and directed towards value.

In this article, value describes how a health system's resources and capacity achieve WHO's framework goals: improved, equitable health, responsiveness, social and financial protection, and efficiency (1). This definition is broader than the economic perspective of health outcomes achieved per unit of cost (4), and aligns with the European Expert Panel's multidimensional concept of value, which encompasses personal, technical, allocative, and societal dimensions (5). According to the WHO framework, building blocks are system inputs, while goals are outcomes. Capacity is necessary but not enough for value, which depends on directing capacity through funding, provider payments, and results measurement. Assessing value needs indicators for each goal, as in Table 1. Kuwait can report some indicators at an aggregate level, but most require linked, case-level data, which this article examines.

**Table 1:** Baseline status, proposed policy actions and value indicators across the six WHO health-system building blocks.

Health-system building block	Baseline status	Proposed policy action	Value indicators	Lead institution and time frame
Service delivery	Three-tier network Capacity expanded 2011–2022 About 85% of spending curative	Rebalance towards primary care and defined NCD pathways	NCD control rates, incidence, and mortality Avoidable admissions Patient-reported experience	MOH (primary care & public health) Medium term
Health workforce	Nationals about 75% of dentists, 41% of physicians, and 5% of nurses Analytic cadres lacked career pathways	Career pathways in informatics, biostatistics and health economics	Analytic posts created and filled Planning decisions supported by routine analysis	MOH & Civil Service Commission Medium term
Health information	Electronic records in primary care Hospital and private integration incomplete	Individual-level coded reporting Case-level costing Data linkage National institute for health information	Share of encounters with coded, linked case-level records Hospitals with case-level costing	MOH (National Health Information Centre) Reporting mandate—near term; Data linkage—medium term; Institute establishment—long term
Medical products	Registration and quality assurance established No national generics policy	Generics policy Value-based tendering and generic substitution	Generic share of volume and spend Prices against GCC benchmarks	MOH (procurement) Near to medium term
Financing	High public share and low out-of-pocket spending Block budgets 100 KWD contribution without indexation	Periodic premium review Case-mix-adjusted allocation Strategic purchasing HTA	Out-of-pocket and catastrophic spending by income group Contribution revenue against cost of care Cost per case	MOH & Ministry of Finance Review near term, allocation and HTA medium term
Governance	MOH as funder, provider and regulator Accreditation results unpublished	Clarify insurance regulation Distinct regulatory function with published accountability	Accreditation and performance results published	MOH (leadership) Longer term

Baseline status based partly on the 2018 Kuwait health system review (9). GCC: Gulf Cooperation Council; HTA: health technology assessment; KWD, Kuwaiti dinar; MOH, Ministry of Health; NCD, non-communicable disease.

Several of Kuwait's neighbours have begun to separate the financing, provision and regulation of care (6–8). In our view, Kuwait's key issue is not which institutional model it adopts but whether the model can measure cost, need, and outcomes at the patient level. Structural reform without adequate analytic capacity risks changing only the system's architecture without improving results. Three gaps remain: limited data infrastructure for decisions, a service mix still focused on curative care, and a budget process not linked to cost or outcome data.

This article examines Kuwait's health system through the WHO health system framework and proposes a reform agenda. First, it argues that Kuwait should develop case-level data alongside institutional reform to ensure new structures are accountable. Second, drawing on recent reforms in Gulf Cooperation Council (GCC) countries, it organises reform options into three interconnected paths: data, financing, and governance.

## Service delivery

Healthcare is organised across six regions in a three-tier system linking primary care with general and tertiary hospitals. From 2011 to 2022, the number of hospitals increased from 14 to 20, beds rose by roughly 30% to 8,735, and 19 primary health centres were added (2). The challenge is therefore one of orientation rather than volume. At the time of the 2018 health system review, about 85% of expenditure remained curative (9), although non-communicable diseases (NCDs) accounted for around 80% of disability-adjusted life years and nearly one in five adults dies from an NCD before age 70 (10). In our view, better value therefore requires stronger primary care, screening and longitudinal NCD management (11).

## Health workforce

Kuwait has expanded its national clinical workforce: Kuwaitis now make up about 75% of dentists and 41% of physicians, but only 5% of nurses (2). However, national recruitment strategies have largely focused on traditional patient-facing cadres, overlooking the specialised human capital required to govern a modern health system. The 2018 health system review found shortages in health informatics, biostatistics, and health economics, and noted that these disciplines lack defined civil service career pathways (9). In our assessment, a specialised analytical workforce is essential; without it, digital infrastructure serves administrative rather than analytical objectives.

## Health information systems

Kuwait has digitised much of its primary care, implementing electronic records and ICD coding (9). However, the gap is not in data collection but in data aggregation: a centralised system that consolidates primary care, hospital, and private-sector records into a linked, case-level resource. We argue that such a system would support routine policy analysis, replacing the current fragmented, facility-specific records and occasional studies (9). Accurate, linked data is crucial for purchasing, premium setting, costing, prevention, and accountability. When such data exists, it is insightful: analysis of 4.4 million claims from the AFYA scheme for retirees revealed that routinely collected administrative records can identify variations in coverage definitions, coding practices, and service rejection patterns (12). However, such analyses are still uncommon and not yet standard practice in planning.

## Medical products and technologies

Registration, licensing, and quality assurance are well established; essential medicines are broadly accessible, and Kuwait purchases part of its requirements collectively through the GCC secretariat, with the remainder tendered nationally and distributed via Central Medical Stores (9). Although Kuwait already purchases generic medicines, the 2018 review found no formal national generics policy to guide substitution, prescribing, or procurement decisions, a gap that may have contributed to growth in the Ministry of Health (MOH) budget (9). Therefore, we argue that reducing this exposure requires either substituting more systematically with generics or building domestic manufacturing capacity, both of which are financing questions as much as procurement ones.

## Health financing

Kuwait performs strongly in financial protection: government sources account for 88% of current health expenditure, the highest share in the GCC, while out-of-pocket spending stands at 10% (13). Yet allocation remains input-based. Providers receive block budgets rather than payments adjusted for risk, activity or outcomes, and full costing and health technology assessment (HTA) are not embedded in coverage decisions (9). Mandatory expatriate health insurance covers about 3.5 million people. After inflation eroded the original KWD 50 premium, Ministerial Resolution No. 306 of 2025 set the standard annual residency contribution at KWD 100, near its inflation-adjusted 1999 level (14, 15). The resolution also introduces exemptions and lower fees for selected groups, moving closer to a graduated approach (15). However, it lacks indexation, so real erosion will resume unless periodic review is institutionalised. Contributions are now partly differentiated, but co-payments remain uniform despite large income differences. Therefore, we argue that case-level cost data are needed to align revenue, contribution design and the actual resource cost of care.

## Leadership and governance

The 2018 Kuwait health system review found that the MOH remains funder, provider, and regulator, a concentration associated with overlapping responsibilities and weak evidence use in decision-making (9, 16). Governance is dispersed across health regions, and insurance oversight sits partly outside the health sector (9); under Ministerial Resolution No. 306 of 2025, insurers licensed by the Insurance Regulatory Unit now also require MOH approval of their health-insurance products (15). The same review reported limited accountability: accreditation results were not published, and providers that failed to meet standards faced no penalties (9).

## Regional reform context

Bahrain, Saudi Arabia, and Abu Dhabi are considered because they share Kuwait's public financing, large expatriate populations, historically dominant health ministries, and movement towards separating financing, provision, and regulation. Because these reforms are recent and not yet evaluated, they are illustrative rather than established models for Kuwait.

Bahrain has introduced a national health insurance scheme for citizens and residents (6). Saudi Arabia is separating regulator, provider and funder roles through health clusters, although implementation remains recent and has not yet been independently evaluated (7). Abu Dhabi illustrates the value of a specialised purchasing function, having established a dedicated group purchasing organisation for its public provider network, whereas Kuwait, Oman, Bahrain and Qatar continue to procure through centralised national tenders (8). In our view, information and institutional reform should advance together: Kuwait should not wait for perfect data before changing purchasing, financing or regulation, but every institutional change should be accompanied by funded, standardised and linked measurement capacity. In our judgement, the main implementation risk is the reverse ordering: new structures being introduced before the data needed to support them are in place.

## Discussion

We propose three mutually reinforcing reform paths. Data governance is listed first because it supports monitoring and evaluation of the other two, not because they should await it. The MOH can mandate individual-level ICD reporting, procedure and laboratory coding, progression towards ICD-11, case-level costing, and linkage across primary, hospital and private-sector data (14).

Measurement is valuable as it informs decisions. Linked case-level data would let the MOH allocate budgets by need and case-mix, benchmark providers on outcomes and cost, purchase services against explicit quality and price criteria, align premiums with the actual cost of care, and evaluate the effects of reform. In their absence, such decisions remain anchored in inputs and precedent.

Therefore, establishing an independent national health information institute, consistent with Kuwait's research agenda and Health Pillar White Paper, would give this work a permanent mandate; the research agenda explicitly proposes upgrading the existing National Health Information Centre into an independent institute (17, 18).

The second path involves financing guided by relevant information. Block budgets tend to reward capacity rather than outcomes. Because the MOH both funds and provides most care (9), purchasing remains implicit rather than contractual. Strategic purchasing (19) would make this process explicit by adjusting budgets for case-mix, linking a portion of funding to quality and outcomes, and publishing transparent accounts of expenditures and results. These measures should balance three objectives: sustainability, achieved through periodic indexation; equity, ensured by graduated contributions; and financial protection, where Kuwait already performs well and which should be preserved. Changes to contributions or co-payments should keep out-of-pocket spending low, particularly for lower-income residents.

Premiums should be indexed and reviewed every three to five years, with protections for lower-income contributors (14). Once case-level costs are available, budget allocation can reflect case-mix, patient complexity and outcomes, while health technology assessment can support explicit coverage decisions rather than implicit rationing.

The third path is governance. Kuwait needs clearer health insurance regulation (6, 9), value-based tendering (20) and generic substitution (9, 21), recognition of informatics and health economics as career pathways (9), and a redirection of delivery towards primary care and defined NCD pathways (9, 11).

Implementation should proceed progressively within each path. In the near term (1–2 years), the MOH can operate within its existing authority by mandating coded case-level reporting from public facilities, institutionalising periodic premium reviews, and piloting case-level costing in selected hospitals. In the medium term (2–5 years), case-mix information may inform hospital budget allocations, an HTA function can be established, and analytic career pathways can be developed in collaboration with the Civil Service Commission (CSC). Longer-term reforms, such as establishing a distinct regulatory function and integrating private-sector data, will likely require new legislation and coordination with the Ministry of Finance. The main constraints include legal mandates such as CSC regulations, data-protection requirements and analytic capacity itself.

We recognise that this argument has limitations. Most of the assessments we rely on were conducted before the current reforms, and the regional reforms are too recent for their outcomes to be judged. Measurement also adds costs and burdens for clinicians. Payment based on case-mix or outcomes can lead to upcoding and selective admission; linking patient records raises privacy issues, and publishing performance differences may be resisted. Other approaches are also defensible, such as separating functions first, as Saudi Arabia has done, or keeping reform minimal because access is already good in Kuwait. We think that, given this coverage and capacity, adding more facilities and staff will add less value than understanding what existing services achieve. This is a judgement, and we would need the data we are proposing to test it.

Kuwait has built capacity, access and financial protection. What remains is the ability to show what that capacity produces. Surveyed Kuwaiti policymakers rated the value of research highly but reported only limited use of it in policy development, and more than half reported no access to the research resources they needed (22). Closing that gap is a precondition for judging reform by value rather than by cost alone.
